# Informal Human Milk Sharing: The Experiences and Practices of Informal Human Milk Sharing in Ireland

**DOI:** 10.1111/mcn.70215

**Published:** 2026-07-02

**Authors:** Niamh Vickers, Anne Matthews, Gillian Paul

**Affiliations:** ^1^ School of Nursing, Psychotherapy and Community Health, Glasnevin Campus Dublin City University Dublin Ireland; ^2^ School of Nursing, Midwifery and Health Systems, Health Sciences Centre University College Dublin Belfield Dublin Ireland

**Keywords:** donors, experiences, human milk, informal human milk sharing, Ireland, practices, recipients

## Abstract

Informal human milk sharing (IHMS) is a contemporary and evolving infant feeding practice. In the qualitative phase of a sequential explanatory mixed methods study, this research explored the experiences, practices, and perceived psychological and emotional impacts of IHMS from the perspectives of donors and recipients in Ireland. Fifteen participants were interviewed: ten donors, three recipients, and two who were donors and recipients. The study is reported according to the standards for reporting qualitative checklist (SRQR). Utilizing reflexive thematic analysis, five themes were identified: (1) Motivations for IHMS, (2) Formal milk banking: invisible, rigid and faceless, (3) The healthcare professional paradox, (4) Navigating risk and safety assurance by the “wee subculture,” (5) Emotional and psychological impact of IHMS. Our findings reveal that participation in IHMS was propelled by personal imperatives and contextual motivations. Formal human milk bank's (HMB's) were described as rigid, invisible and “*quite the effort.*” Participant narratives depict a culture where CMF was “*pushed left, right and centre”* in maternity settings as well as the intentional concealment of IHMS from healthcare professionals. Safety and risk mitigation were framed through “*a huge trust network.*” IHMS has profound emotional and perceived psychological outcomes that were represented as a “*light switch*” release of maternal stress and anxiety for recipients while fostering a sense of pride and a “*nice, warm, fuzzy feeling”* for donors. There is an imminent need for policy reform and robust clinical frameworks to facilitate healthcare professional engagement, risk mitigation and enhancement of the visibility and accessibility of formal HMBs. Future research should evaluate HCP understanding of HMBs and IHMS in Ireland and similarly under‐researched regions.

## Summary

- This research constitutes the first qualitative investigation of informal human milk sharing (IHMS) in Ireland and provides a rich exploration of the experiences, practices and perceived psychological and emotional impacts of IHMS from the perspectives of donors and recipients.
- Engagement with IHMS had profound emotional and perceived psychological outcomes which were represented as a distinct resolution of maternal stress and anxiety for recipients while fostering a significant sense of pride and fulfillment for donors.
- Participant perspectives highlighted a systemic invisibility of formal human milk bank (HMB) systems and a dominant commercial milk formula (CMF) culture within maternity settings. Addressing these disparities requires policy reform and healthcare professional (HCP) education. This could facilitate HCP dialog and mitigate risk while enhancing the visibility and accessibility of formal HMBs.

## 1 Introduction

Breastfeeding is the safest and most efficacious method of infant feeding (Ahmad and Haque 2025). Accordingly, it has been opined that breastfeeding can be conceptualized as an infant's first vaccine, conferring immediate protection from disease with enduring advantageous effects (World Health Organization 2025). Central to these benefits is the provision of human milk (HM), a complex, dynamic and unparalleled substance comprising a comprehensive nutritional profile and substantive bioactive components (Szyller et al. 2024; Pérez‐Escamilla et al. 2023). These bioactives contribute fundamentally to infant health, development and immune‐stimulating functions (Szyller et al. 2024). A proliferation of scholarly evidence continues to reveal emergent advantages of HM which strengthens its role as a dynamic biological system that offers substantive benefits. Further, the utilization of HM also bestows advantageous health, societal, environmental and economic outcomes (Vickers et al. 2024). Consequently, global health organizations recommend breastfeeding initiation within the first hour of life, exclusive breastfeeding for 6 months and continued breastfeeding until 2 years and beyond, along with complementary foods (World Health Organization 2023). Global exclusive breastfeeding rates within the first 6 months stand at 48%, which is a discernible gap from the World Health Assembly target of 60% by 2030 (World Health Organization 2025). In instances where mothers' own milk (MOM) is not available, global health organizations recommend providing donor human milk (DHM) from a milk bank, not commercial milk formula (CMF) until MOM is obtainable or breastfeeding has been established (PATH 2017). An emerging field of research demonstrates that direct breastfeeding confers enhanced benefits for the maternal and infant dyad (Coutsoudis et al. 2026). HM composition and caloric content are dynamic and systematically dependent on many factors, including circadian influences (Woortman et al. 2025; Italianer et al. 2020), environmental factors such as storage and handling (Slater et al. 2024), and thus expressed human milk (EHM) may vary biochemically from breastfeeding. Further, the activation of metabolic pathways during breastfeeding through the interactions between infants saliva and HM and the establishment of the infant's oral and gut microbiota further empirically affirm its enhanced benefits (Sweeney et al. 2018). Breastfeeding is also associated with reduced postpartum depression, anxiety, cardiovascular disease, diabetes, breast and ovarian cancers (Coutsoudis et al. 2026).

It has been postulated that CMF is inferior to the various alternatives in the HM continuum, including EHM, DHM, and wet nursing (Ahmad and Haque 2025). The global network of HMBs has increased significantly in recent years, driven predominantly by the recognition of the importance and value of DHM (Lahav and Harison 2025). Globally, there are approximately 800 operational HMB facilities across 66 countries (Belekar 2025). HMBs are instrumental in the collection, screening, processing, pasteurization, storage, quality control, and safe dispensing of DHM to infants in the absence of MOM (Arslanoglu et al. 2023). It is widely acknowledged that HMBs play a significant role in the promotion, protection, and support of breastfeeding (Fonseca et al. 2021). As such, HMBs have been asserted as vital pillars in neonatal public health (Machado et al. 2025). Moreover, recent findings affirm that the receipt of DHM has further positive implications that transcend infant health outcomes and positively impact parental wellbeing by reducing anxiety and enhancing mental health (Brown et al. 2024). Nevertheless, DHM is not universally available to all infants when required and global efforts are necessitated to expand HMBs worldwide (Israel‐Ballard et al. 2024). In Ireland, there is one HMB located in County Fermanagh, Northern Ireland. This facility has been in operation for 26 years and provides DHM to 27 hospitals across Ireland. In 2024, they distributed DHM to 700 and 83 premature infants in Ireland (Western Health and Social Care Trust 2026). Nevertheless, due to the lack of universal accessibility of DHM, there is evidence that some individuals engage in HMS as an alternative option for supplementation provision when MOM is not available (Vickers et al. 2024; Li et al. 2025).

A recent mixed‐methods systematic review (MMSR) highlighted the imminent demand for in‐depth investigations of the experiences, practices and psychological impacts of IHMS (Vickers et al. 2024). Accordingly, a sequential explanatory mixed methods study was planned to explore IHMS within the geographical context of Ireland due to an absence of empirical research within this geographical context. This current study constitutes the qualitative phase which offers a pioneering contribution that explores the experiences, practices and perceived psychological and emotional outcomes of IHMS in Ireland. This study utilizes reflexive thematic analysis to provide a deep and interpretive understanding of the nuanced realities of IHMS from the perspectives of donors and recipients in Ireland.

## 2 Methods

### 2.1 Aim

The aim of this study was to explore and gain an in‐depth understanding of the experiences and practices of IHMS among donors and recipients in Ireland.

### 2.2 Research Design

As part of a larger mixed‐methods explanatory sequential design, this phase utilized a descriptive, inductive methodology to bring meaning to the phenomena of IHMS and the subjective views of participants. The interview guide/protocol was developed to build upon and explain the findings of a preceding phase, a quantitative national survey of IHMS donors and recipients in Ireland (Vickers et al. 2025).

### 2.3 Sample and Recruitment

Participants who indicated their interest in participating in interviews were recruited from an expression of interest form included in the initial phase of this study. The eligibility criteria for study participants were: (1) aged 18 years or older; (2) participated in IHMS either as a donor or a recipient within Ireland in the last 18 months (3) participated in the initial quantitative phase of the study.

### 2.4 Data Collection

The interviews were conducted via the Zoom online platform. The duration of the interviews varied between 38 and 105 min.

### 2.5 Data Processing and Analysis

The collected interview data were transcribed verbatim and cross‐referenced for accuracy by comparing them to the original recordings. Data were imported to NVivo (version 15) for analysis. The data were analyzed inductively utilizing reflexive thematic analysis, drawing on the six‐phase framework originally developed by Braun and Clarke in 2006. This study adheres to the refined six‐phase framework as detailed in Table 1 (Braun and Clarke 2022).

**Table 1:** Thematic analysis process following the approach of Braun and Clarke (2022).

Phase		Application of steps
1	Familiarization with data	Data were imported to NVivo (version 15). Deep immersion included reading transcripts, taking reflective and notes relating to the raw data, and listening to audio‐recordings.
2	Generation of initial codes	An initial round of inductive coding was conducted by systematically engaging with each transcript line by line within NVivo (version 15) software. An initial 96 codes were generated at this phase.
3	Generating initial themes	These initial 96 codes were grouped and collated into potential themes by utilizing visual maps to explicitly view and organize patterns of meaning across the dataset. The generation of themes was an interpretive process constructed by the researcher based on the data, the researchers conceptual insights and the research questions,
4	Development and reviewing of themes	Candidate themes were visualized, reviewed and refined. During this phase the data were revisited to ensure that the named themes were distinct and coherent and represented distinct patterns of shared meaning and coherent across the dataset. This refinement process and the generation of themes ultimately led to the consolidation of the themes into a final set of five.
5	Refinement and naming of themes	The final set of themes were defined and named. This process involved writing a detailed narrative for each theme ensuring each theme was clearly delineated.
6	Writing the report	This involved finalizing, refining and integrating the selected data extracts with a rich, comprehensive and interpretative narrative that connected the findings to the literature and research question.

### 2.6 Rigor

This study adheres to the Standards for Reporting Qualitative Research guidelines (O'Brien et al. 2014). This study incorporated PPI engagement which further strengthened its rigor. Participant feedback was utilized during data collection to ensure accuracy of accounts (Tracy 2010). Reflexivity was integrated as an “*ongoing process of reflection”* throughout the research process (Braun and Clarke 2022, p. 18). The primary researcher (NV) is a PhD candidate, midwife, nurse, lactation consultant (LC), mother of two children with prior, personal experience of breastfeeding but not IHMS. Analysis involved collaborative reflexivity with PhD supervisors (GP, AM) who possess extensive qualitative and shared disciplinary expertise. This collaborative dialog, supported by reflective journaling and an audit trail ensured analytical depth (Olmos‐Vega et al. 2023). The incorporation of pseudo‐anonymized extracts from the data using pseudonyms to illustrate the findings and provide empirical support for the authors' interpretive commentary.

### 2.7 Ethics and Public and Patient Involvement (PPI)

Ethical approval was sought and granted to conduct this study by Dublin City University Research Ethics Committee (DCUREC/2024/083) on the 21st May 2024. An interview guide/protocol was developed based on the analysis of the quantitative findings from the survey (Vickers et al. 2025). To ensure relevance and sensitivity, the interview guide was refined through PPI with two individuals who provided lived experiences of IHMS from donor and recipient perspectives, respectively. Their feedback was instrumental in capturing the nuances of IHMS. Two pilot interviews were conducted to evaluate flow, clarity, and effectiveness of the interview guide/ protocol.

## 3 Results

**Figure 1:**
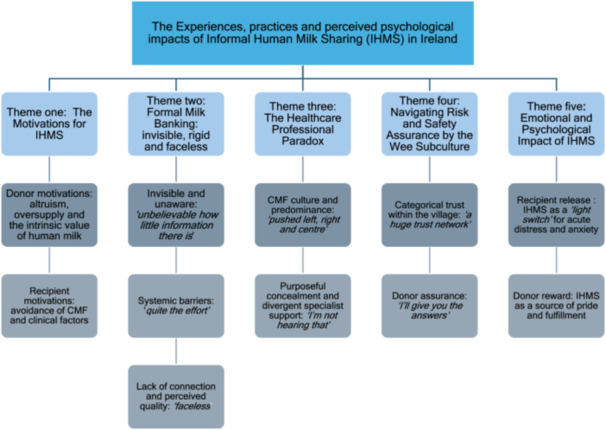
Reflexive thematic analysis: themes and subthemes.

A total of 15 participants, of which 10 were donors, 3 were recipients and 2 who had engaged in IHMS as both donors and recipients were recruited for the study. The majority of participants (*n* = 10) were between 3 and 39 years of age, with the remaining (*n* = 5) aged 40 or over. The majority of participants had engaged in IHMS within the last year (*n* = 8). From a geographical perspective, participants lived in two provinces in Ireland: Leinster (*n* = 10) and Ulster (*n* = 5), with (*n* = 4) participants in Ulster residing in counties in Northern Ireland. The demographic characteristics of study participants are detailed in Table 2. Five predominant themes were identified from the analysis of the data and are visually presented in Figure 1. Each theme had a number of subthemes which will be detailed comprehensively below. An overview of the five themes and associated subthemes is provided in Table 3.

**Table 2:** Demographic characteristics of participants in semi‐structured interviews.

Characteristics	*n*	(%)
Participant category		
Donor	10	(66.6)
Recipient	3	(20)
Donor/Recipient	2	(13.3)
Last milk shared		
< 6 months	6	(40)
< 12 months	2	(13.3)
> 12 months	7	(46.6)
Current Age		
30–35 years	4	(26.6)
36–39 years	6	(40)
40+ years	5	(33.3)
Gender		
Female	15	(100)
Number of children		
1	6	(40)
2	4	(26.6)
3	4	(26.6)
4+	1	(6.66)
Ethnicity		
White Irish	13	(86.6)
Other white background	1	(6.66)
Brazilian	1	(6.66)
Education		
Secondary or equivalent NFQ 4–6	2	(13.3)
Degree level or equivalent NFQ 7–8	7	(46.6)
Postgraduate/Masters/Doctorate NFQ 9–10	6	(40)
Province		
Leinster	10	(66.6)
Ulster	5	(33.3)
Employment status		
Employed	8	(53.3)
Fulltime parent	6	(40)
Student	1	(6.66)
Marital status		
Co‐habiting	1	(6.66)
Married	14	(93.3)

**Table 3:** Overview of themes.

Theme name	Associated subthemes	Overview of theme
The motivations for IHMS	Donor motivations: altruism, oversupply and the intrinsic value of human milk Recipient motivations: avoidance of CMF and clinical factors	Highlights the motivations for engagement with IHMS. Donors were motivated to help others in need. This altruistic imperative was often associated with an oversupply of HM and a desire not to waste it. Recipients were motivated by a desire to avoid CMF. Various maternal and infant factors clinical factors were key motivators for engagement with IHMS.
Formal milk banking: invisible, rigid and faceless	Invisible and unaware: ‘*unbelievable how little information there is’* Systemic barriers: *‘quite the effort’* Lack of connection and perceived quality: *‘faceless’*	Explores the perceived invisibility of HMBs particularly among HCPs. It highlights the reported barriers and inhibitors to the engagement with HMBs. It explores the reported lack of personal connection and a perception of reduced HM quality with HMB donation.
The healthcare professional paradox	CMF culture and predominance: *‘Pushed left, right, and centre’* Purposeful concealment and divergent specialist support: *‘I'm not hearing that’*	Highlights the paradoxical role of HCP's who were described as barriers to breastfeeding through a perceived CMF culture, yet in rare cases functioned as facilitators of IHMS. The intentional concealment of IHMS from HCP's was common among participants.
Navigating risk and safety assurance by the ‘wee subculture’	Categorical trust within the village: *‘a huge trust network’* Donor assurance: *‘I'll give you the answers’*	Explores how risk and safety are constructed by IHMS participants. Safety and trust are framed through a ‘wee subculture’ which was depicted as a ‘huge trust network’ where its members have shared values and a collective belief in the significant value of HM. A sense of safety was established by donor transparency, narrative interactions and the in‐person exchange of HM.
Emotional and psychological impact	Recipient release: IHMS as a *‘light switch’* for acute distress and anxiety Donor reward: IHMS as a source of pride and fulfillment	IHMS was associated with positive psychological and emotional outcomes for IHMS participants. The instantaneous reduction in stress and anxiety on receipt of HM was described by recipients and donors described a sense of pride and fulfillment.

### 3.1 Theme 1: The Motivations for IHMS

This theme underscores the imperatives of engaging in IHMS from both donor and recipient perspectives. These were underpinned by powerful personal imperatives that were interlinked with factors such as necessity, inherent values and practicality which propelled them to participate in IHMS.

#### 3.1.1 Donor Motivations: Altruism, Oversupply and the Intrinsic Value of Human Milk

The central motivation for donors to engage in IHMS was articulated as a form of altruism: “*Wanting to help other mammies, parents, caregivers. just wanting to help”* (Sinéad). One participant stated that her ability to donate stemmed from her own breastfeeding journey which transformed personal gratitude into an enabling imperative to support those struggling: “*Like I was just so happy that it worked for me, and then so much so that I was able to help other people with it”* (Rebecca). Another echoed this and also positioned the donation as a proud gift, stating: “*Look! I felt great being able to help out. You know the little baby especially, you know, considering mammy wasn't there. like… it is kind of like a gift to somebody.”* (Emma). Many reported they had an oversupply of milk which served as an important trigger to donate: *“…. when you have an oversupply like that, like you have to…. you have to share it.”* (Patricia). This was intertwined with a powerful desire to avoid wasting milk which was denoted as a “*valuable substance”* (Grace) and it would be “*such a waste not to”* (Marie). A parallel, nuanced factor to donate was practical concerns. Specifically, freezers were full and milk was nearing its expiration date, translating their altruistic desire into an urgent requirement: “*It was just stacking up… I was like I need to look about spreading some of this around”* (Patricia).

#### 3.1.2 Recipient Motivations: Avoidance of CMF and Clinical Factors

The avoidance of CMF was a key motive for all recipients often underpinned by their personal beliefs about the physiological impact of CMF on lactogenesis: “*I really didn't want to give formula because I know it would impact my supply…*. (Shauna). Many were driven to provide *‘the best thing’* (Shauna) for their infants highlighting *‘the cold, hard truth of it without hurting anybody's feelings is…. there is nothing as good as breastmilk. formula is second best”* (Caoimhe). Both maternal and infant factors were also key motivators for recipients to seek DHM which primarily occurred when faced with challenges that necessitated the provision of urgent supplemental feeding. Infant factors including dehydration: “*he had, like the brick dust in his urine”* (Caoimhe) and admission to neonatal intensive care (NICU), “*he was very ill”* (Sinéad) motivated recipients to engage in IHMS. Maternal‐related factors were also prominent and included insufficient milk supply, maternal illnesses and complex complications such as postpartum hemorrhage: “*I was clinging onto the walls…I was so dizzy…I felt so rotten after the bleed*.” (Shauna). It was acknowledged that without IHMS recipients faced resorting to CMF*: ‘Because otherwise I had nothing at all like…*.” (Deirdre).

### 3.2 Theme 2: Formal Milk Banking: Invisible, Rigid and Faceless

This theme specifically explores the perceived invisibility, lack of professional awareness of HMBs and pervasive barriers of the system that inhibited engagement by participants.

#### 3.2.1 Invisible and Unaware: “Unbelievable How Little Information There Is”

Several participants noted the significant lack of awareness and invisibility of the formal HMB, particularly among HCPs. Several participants highlighted that their General Practitioners (GPs) were unaware of the HMB, with one acknowledging: *‘it's just unbelievable how little information there is. like the official milk sharing’* (Marie). Another commented that their GP: “*had never heard of the milk bank, and she was certainly like kind of rolling her eyes and saying, I don't know what that is, I want to have nothing to do with it”* (Siobhan).

#### 3.2.2 Systemic Barriers: “Quite the Effort”

Donors cited various barriers, such as medication ineligibility, their milk already being frozen, or missing infant age eligibility windows required by the HMB. Some perceived the commitment to formal donation as a critical barrier: “*it was far too restrictive and demanding to be something I'd even consider”* (Aoife). Others noted the minimum volume requirement as inhibiting: “*they want X amount by a certain date, and I think that's a lot of pressure.”* (Emma). Further logistical factors were described by participants such as unsustainable efforts involving “*months of recording freezer temperatures”* (Siobhan), additional storage requirements, where *“milk needs to be stored separately”* (Fiadh) and strict protocols “it is strict [HMB]…. you're not even allowed to use the sterilizer” Conversely, IHMS had no “*hoops to jump through. There was no trying to schedule blood drawings on a Friday afternoon”* (Siobhan).

#### 3.2.3 Lack of Connection and Perceived Quality: “Faceless”

Donors noted a significant omission in feedback regarding the use of their DHM. Despite the importance donors placed on knowing how their DHM was used, this was not provided by the HMB: “*It would have been nice to know… ohhh, that was used for I don't know 27 babies”* (Siobhan). Another highlighted that this information was once provided, but now omitted due to data protection regulations: “*We used to get a wee note…. your milk has gone to help 3 babies in the NICU…. nowadays they don't even tell you that information”* (Sinéad). Several donors contrasted the perceived *‘faceless’* HMB with the personal connection of IHMS: “*It was a wee bit faceless, whereas, like I do prefer this one”* (Patricia). This was mirrored by others noting: “*You know that a real human being has picked it up and used it”* (Siobhan). Some described a perceived superiority of IHMS, denoting an understanding that pasteurization diminishes quality: “*When the milk goes up North [to the HMB], it's obviously pasteurized. I know that it's not going to be as good”* (Aoibheann).

### 3.3 Theme 3: The Healthcare Professional Paradox

This theme refers to the perceived paradoxical role of HCPs who were described as significant barriers to breastfeeding and yet, in a rarity of cases, as vital facilitators of IHMS. Collectively, participant narratives described a CMF culture within maternity settings. Consequently, there was an intentional concealment of IHMS among participants in healthcare settings.

#### 3.3.1 CMF Culture and Predominance: “Pushed Left, Right, and Centre”

A prominent finding was the perception of a pervasive CMF culture within the maternity settings where HCPs were perceived as being: “*so quick to offer formula… it just seems to be the solution for a lot of healthcare professionals”* (Aoibheann). CMF was the prominent narrative that participants highlighted was “*pushed left, right, and centre”* (Siobhan) and that “*it was all about formula from the start”* (Shauna). This was perceived by participants as a default option rather than the provision of specialist breastfeeding support in a multitude of situations, including maternal exhaustion which was articulated by one participant as a justification for HCP suggesting CMF in her experience: “*I really think you should give a bottle of formula because you're exhausted”* (Aoibheann). One recipient described a “*poor experience”* where she felt she was being “*really talked down to”* by staff who she described insisted, “*your baby is starving…. and you're still refusing formula,”* leaving her in “*floods of tears”* (Shauna). Another shared this sentiment when their baby was in NICU and she described an experience of a HCP saying: “*he's starving, just give a bottle”* (Aoibheann).

#### 3.3.2 Purposeful Concealment and Divergent Specialist Support: “I'm Not Hearing That”

A common practice among this cohort was the intentional concealment or non‐disclosure of IHMS with HCPs. One recipient claimed she had pumped the milk she received through IHMS “*because I don't think that they would approve of it”* (Grainne). Another “*completely lied”* about the source of her milk, reflecting that if she were honest, staff would be like “*Oh, don't… throw it in the incinerator*” (Deirdre). Another noted that despite having a “*supportive”* GP, she would not disclose IHMS as it would be “*opening a can of worms”* (Siobhan). When participants did choose to disclose, some were met with conflicting responses. One participant portrayed differing encounters within the same healthcare setting with different HCPs where the midwifery staff were “*very supportive”* yet, the medical team “*read us the riot act…”* (Caoimhe). Another recalled mentioning IHMS in the NICU only to be met with a refusal of engagement: “*they said… I'm not hearing that. I don't know about that”* (Cara). A paradoxical experience transpired for some participants where a minority of HCPs facilitated IHMS, namely LC's or midwives. One LC actively encouraged a recipient to use a friend's previously expressed colostrum, saying: “*Oh my god, get it!”* (Shauna). Another LC actively orchestrated the entry of informal milk into the maternity site: “*and so I did, I brought it in to the hospital’”* (Aoibheann). This was also evidenced when a homebirth midwife acted as an intermediary for an exchange between her two clients. This direct request to the donor client by the midwife was met with an immediate “*Yeah, absolutely”* (Siobhan).

### 3.4 Theme 4: Navigating Risk and Safety Assurance by the “*Wee Subculture”*

This theme relates to the architecture of navigating risk and safety in the context of IHMS in Ireland where risk was reportedly circumvented and safety was perceived as assured by the “*wee subculture”* of mothers with shared beliefs and values.

#### 3.4.1 Categorical Trust Within the Village: “A Huge Trust Network”

This subtheme exemplifies how safety is purposively anchored by an inherent trust among members of this “*support network”* (Sinéad) or “*little village in the background”* (Fiadh) which forms a “*little, tiny, niche part of their life”* (Caoimhe). The village is multidimensional in nature comprising many social layers where participants engage in IHMS with friends, acquaintances, family members, personal networks and connections made in online platforms. Collectively, this group is a “*wee subculture….a wee clique”* (Patricia) and belonging to this establishes “*a huge trust network”* (Sinéad). These social layers enable participants to attain a sense of safety through transparency, connection, and a shared moral code whereby “*everybody is very careful…. the community is quite strong”* (Cara). At the crux of this symbolic village, trust also emanates from the collective understanding of the formidable value of HM. Participants portrayed HM as “*miraculous”* (Aoife), “*primal”* (Marie), and “*the most important thing”* (Cara) which is incomparable to “*anything else”* (Grainne). For many participants, an inherent safety assurance is that the donor is breastfeeding their own child: “*I can feed my own kids…. How much harm can it do to feed someone else with that milk?”* (Siobhan). Trust is actively constructed through narrative exchanges, dialog between online connections and face‐to‐face exchanges which establishes a “*good rapport”* (Rebecca), signifying that this dialog and trust is a form of “*vetting”* (Sinéad, Deirdre).

#### 3.4.2 Donor Assurance: “I'll Give You the Answers”

This subtheme relates to how donors take a proactive role in facilitating transparency by providing contextual information about themselves and their milk. A prominent finding was that donors were aware of the sensitive nature of having to request milk and the potential “*emotional state”* (Aoife) of the recipient. Donors understood that recipients might feel a “*sense of like beggars can't be choosers”* (Aoife) or fear that asking questions might imply a lack of gratitude: “*if I ask them questions. they'll think I don't want their milk”* (Sinéad). Donors circumvented this barrier by proactively “*giving the answers in case you're too embarrassed to ask me yourself”* (Sinéad). By providing information “*off the bat”* (Rebecca), they removed the burden of vetting from the recipient to alleviate “*anxiety”* (Rebecca). Information regarding diet, alcohol and medication was shared to “*leave the ball in their court”* (Caoimhe) so that the recipient could “*make a choice”* (Caoimhe) whether to “*accept or not”* (Sinéad).

### 3.5 Theme 5: Emotional and Psychological Impact of IHMS

This theme explains the perceived psychological and emotional impact of IHMS on donors and recipients. The exchange is acknowledged as “*symbiotic”* (Patricia), where both donors and recipients acquire positive emotional outcomes as a result of IHMS.

#### 3.5.1 Recipient Release: IHMS as a “Light Switch” for Acute Distress and Anxiety

Recipients described how the prelude to needing milk was often fraught with acute distress emanating from circumstances, including the physiological sequelae of postpartum hemorrhage*: I had a postpartum hemorrhage after the delivery…. I had like a really low blood pressure, “a temperature of 39.2”* (Shauna) or delayed lactogenesis following a cesarean section where it: “*takes extra time for the milk to come in”* (Caoimhe). These eventualities caused significant negative emotions, leaving participants “*absolutely, emotionally wrecked”* (Sinéad), “*overwhelmed”* (Deirdre) and “*so stressed and worried”* (Grainne). Subsequently, the provision of milk via IHMS created a pivotal transition in the psychological state of many recipients. Metaphorically, participants articulated the receipt of DHM as a “*light switch”* (Sinéad) which instantaneously resolved acute distress. One recipient observed the physiological influence of this relief, stating that once she had the “*buffer”* of DHM, her own supply “*just kept flowing, kept flowing, kept flowing”* (Sinéad). Another highlighted it provided: “*a huge sense of relief of calmness”* (Grainne). Ultimately, recipients described this as an “*amazing”* bridge that “*got us over the hump”* (Shauna) and resulted in positive emotional sentiments including feeling “*proud”* (Shauna, Deirdre, Sinéad) and “*grateful”* (Grainne, Caoimhe).

#### 3.5.2 Donor Reward: IHMS As a Source of Pride and Fulfillment

Donors articulated powerful reflections regarding an intense feeling of pride where some were “*proud that I was able to make that milk and give it to them”* (Caoimhe), attributing this affect to the “*hard work”* afforded to pumping (Emma). One donor likened IHMS to blood donation, elucidating the intrinsic pride derived from the body's ability to produce a life‐giving substance: “*you're giving something that your own body has produced…. there's a certain pride”* (Siobhan). Donors conveyed that IHMS was such an *‘emotional thing’* (Cara, Fiadh) that resulted in “*innate appreciation”* (Grace) and immense “*gratitude”* (Fiadh, Cara, Aoife). Symbolically, participants depicted the emotional aftermath as “*a nice, warm, fuzzy feeling”* (Sinéad) that “*makes you feel so good”* (Patricia).

## 4 Discussion

This empirical study has elucidated the lived experiences, practices and perceived psychological and emotional impacts of IHMS in Ireland from both donor and recipient perspectives. Fundamentally, the belief in the inherent and superior value of HM engenders a deeply upheld desire to engage in IHMS. The altruistic desire of donors was underpinned by a deeply positioned, empathic and sensitive understanding of recipients' needs. The realities of an oversupply and excess milk accumulation, as well as an innate resistance to wasting HM prompted donors to engage with IHMS. These underpinnings align with conceptualizations of HM donation as a transcendence of intensive mothering and care work which are profoundly intertwined in maternal identity (Oreg 2023). Explored extensively by Hays (1996), intensive mothering pertains broadly to ideology that motherhood is both instinctive and innate where mothers should selflessly afford substantive commitment and dedication to the upbringing of their child(ren). Recently, the commitment to this ideology is evident within breastfeeding discourses (Verniers et al. 2022; Oreg 2023). The notions of “care work” epitomize the unpaid, often invisible and labor‐intensive activities associated with motherhood including breastfeeding, expressing milk, and donation (Carroll 2015; Oreg and Appe 2022). Consequently, it has been suggested that HM donation enables donors to extend their nurturing and mothering beyond their family. Similarly, our study revealed that recipients were motivated to seek milk via IHMS due to the deeply held desire to avoid CMF and provide HM which resonates with contemporary ideals of motherhood. Coupled with this, there were often complex clinical factors which necessitated urgent supplemental feeding. These motivations are now well‐established and substantiated drivers of IHMS within both the Irish and international literature (Vickers et al. 2024, 2025; Peregoy 2021; Li et al. 2024; Mampane and Wolvaardt 2024; Li et al. 2025).

Our findings depict an interesting discourse of the formal HMB system within Ireland which delineates pervasive barriers, professional invisibility and a “*faceless”* experience when compared to IHMS. Indeed, multifaceted practical, logistical and regulatory barriers detracted from engagement with formal systems. In a recent survey of IHMS participants in Ireland, 87.6% considered donating to the HMB; however, only 30.1% progressed through the screening to attain an approved donor status (Vickers et al. 2025). This current study augments this actuality and posits that prominent contextual barriers inhibit the engagement with HMBs. These barriers are comparable with findings from other empirical work which explored the barriers to HMB donation in the UK and US (Dos Santos et al. 2024). Analogous to this is the affirmation that these pervasive barriers to HMB donation function as enablers to the engagement with IHMS which was asserted in a recent MMSR (Vickers et al. 2024). A key finding in this study was a reported lack of awareness of the HMB among HCPs by participants which is consistent with previous work in other jurisdictions (Dos Santos et al. 2024; Mathias et al. 2023). This highlights the imperative need to increase awareness of HMB among HCPs, as it has been ascertained that HCPs play a fundamental role in shaping insights and an imperative role in promoting formal pathways (Adu‐Kofi et al. 2025; Monti et al. 2024; Mahdikhani et al. 2025). This study noted that some donors were propelled to engage in IHMS rather than an HMB due to the perception that mandatory pasteurization in HMBs diminishes distinct and valuable properties of HM, a finding that has been previously elucidated by others (Perrin et al. 2016; Mathias et al. 2023). In this study, HMBs were depicted as “*faceless”* and there was a preference for IHMS due to personal connection with recipients. This aligns with findings from a recent review which established this as a psychological barrier to donation to HMB (Monti et al. 2024). Importantly, perceptions of the HMB were consistent across geographical locations and did differ between those residing in Northern Ireland or the Republic of Ireland.

Findings from our study describe a perceived CMF culture within maternity settings. This phenomenon has been previously documented within the study's geographical context where mothers described the emphasis on CMF in the maternity ward as pressuring and traumatic (Lawlor et al. 2023). In our study, participants consistently articulated that recommendations for CMF transcended medical necessity which is in direct contravention to national and global standards that assert that breastfed infants should not be provided with any food or fluids other than breastmilk, unless medically indicated (World Health Organization 2018; Health Service Executive 2022). This is a salient finding due to the substantive evidence affirming that the introduction of CMF is a categorical risk factor for reduced breastfeeding duration and exclusivity (Pérez‐Escamilla et al. 2022). Notably, another recent study evaluated local guidelines pertaining to medical indications for supplementation in breastfed infants in the 19 maternity units and two tertiary neonatal units in Ireland and their findings confirmed that 43% of settings did not have guidelines in place and those that did were not in accordance with international recommendations (Almaiman et al. 2025. There was a notable preponderance of covert concealment and non‐disclosure of IHMS to HCPs described by participants. This is corroborated by further evidence noting the absence of acceptance of, support for, or input from HCPs pertaining to IHMS (Vickers et al. 2024; Perrin et al. 2016; Onat and Karakoç 2019; Vickers et al. 2025). However, these findings are fundamentally divergent from recent scholarly work undertaken in New Zealand which found that IHMS is highly supported by HCPs (Harris et al. 2024). It is argued that this variance may be attributed to the formalized recognition of DHM and IHMS by the New Zealand College of Midwives in their consensus statement on DHM and milk sharing which articulates that HM from a donor mother is a suitable substitute in the absence of MOM and provide guidance and practice recommendations to support women, HCPs and maternity services in informed decision making pertaining to ethical and safe milk sharing practices (New Zealand College of Midwives 2019). This cross‐cultural divergence suggests that the non‐engagement or support for IHMS by HCPs is not a conclusive feature of IHMS globally and that geographical disparities exist as a consequence of local, cultural and health system factors. Indeed, with a lack of national policy or guidance pertaining to IHMS in Ireland, it is argued that the non‐engagement by HCPs in IHMS is an expected eventuality given the medico‐legal constraints faced by HCPs in the absence of guidance. There are risks involved in IHMS, by virtue of the exchange of HM which is a complex bodily fluid. The significant risks asserted include the transmission of disease, contamination with unsafe substances, and bacterial contamination during expressing and handling (Blackshaw et al. 2021). A salient paradox to this widespread non‐engagement is the occasional HCPs, namely specialist LCs or midwives, who functioned as facilitators of IHMS concurs with previous work (O'Sullivan et al. 2016; Perrin et al. 2016; Vickers et al. 2024).

Trust and safety within this geographical context are inextricably embedded within a trust‐based system, a “*wee subculture”* which acts as the perceived safety net. Inherent to this, is the reality that donors in Ireland assume this role by proactively giving the information to recipients, an act which is deeply rooted in empathetic sensitivity and a finding consistent with recent scholarly work in Hong Kong (Li et al. 2025). This finding concurs with recent scholarly work in Hong Kong, where it was ascertained that donors proactively provide information to recipients voluntarily (Li et al. 2025). Open dialog and in‐person exchanges are regarded as a substitute for the clinical screening processes associated with the formal HMB systems (McCloskey and Karandikar 2018; Onat and Karakoç 2019. These lay screening practices are in contrast to those in formal HMB systems. Consequently, IHMS has been conceptualized as a profound display of resistance to the medical discourses of HM that views expressed HM as a dangerous biosubstance and a potential biohazard that should not be shared (Palmquist 2015). Contrastingly, for IHMS participants, HM was viewed as “*miraculous”* with intrinsic value ascribed to this “*valuable substance,*” and so, the practice unequivocally transcends the simple exchange of HM to a deeply emotive and symbiotic experience for donors and recipients with significant trust. This resonates with Shaw (2019), who asserted that IHMS participants assign a substantive value to HM and the act of donation constructs networks of bio‐intimacy evidenced in the establishment of relational and caring bonds through the sharing of this biological material. Importantly, the shared values, beliefs and experiences of IHMS participants facilitates a “*huge trust network”* and collective solidarity in this “*wee subculture.”* Indeed, it has been clearly articulated that milk exchange is symbolic of solidarity involving mutual recognition and a collective commitment to addressing common concerns and enhancing health (Monti et al. 2024; Oreg 2025). This can be conceptualized by drawing on Oreg (2025) exploration of solidarity among donors and communities in HM banking who affirmed that shared experiences and shared identities rooted in motherhood foster coalescence, unity and collaboration among its actors and thus the shared commitment to infant wellbeing is the underpinning for solidarity. By applying Oreg (2025) lens to this study, it is evident that the collective solidarity within the IHMS participants is akin to that evidenced in formal systems. Indeed, the shared experiences, shared identities of motherhood, and the collective action of the key actors through the “care work” of donating and similarly in the pursual of HM by recipients with the collective endeavor of enhancing the wellbeing of infants provides the foundation of solidarity in this context.

The emotional and psychological impact of IHMS on mothers is an evolving theme within the contemporary landscape of IHMS discourses and scholarship which has highlighted that psychological benefits can be attributed to the engagement with IHMS (McCloskey and Karandikar 2019; Wagg et al. 2022). Hence, this study provides valuable insights into the profound impact of IHMS on the emotional and psychological wellbeing of IHMS participants in Ireland. Our findings portray the unambiguous influence IHMS had on recipients who journeyed a trajectory of acute distress and anxiety in the prelude to IHMS to a categorical release of these deleterious emotions and resolution of their distress. Fundamentally, the receipt of milk via IHMS was conceptualized as a “*light switch*” release denoting the immediate resolution of distress. The instantaneous transition was palpable among all recipient participants and some noted that the receipt of milk enabled their own milk to flow. Indeed, this aligns with empirical evidence which affirms that heightened psychological distress can interrupt milk flow and subsequently impact milk supply (Mohd Shukri, et al. 2018; Nagel et al. 2022). Likewise, donors revealed resounding positive psychological impacts which were ascribed to IHMS, such as pride, fulfillment, appreciation, and gratitude. Collectively, IHMS was characterized as symbiotic which represents a mutual psychological gain for both donors and recipients.

### 4.1 Strengths, Limitations, Implications

This study possesses several notable strengths that make a significant and novel contribution to the field of IHMS. Foremost, it represents the first qualitative exploration of IHMS conducted in Ireland, offering a nuanced understanding of the practices and experiences of donors and recipients in Ireland. Methodologically, the incorporation of PPI and a pilot phase was a notable strength. The application of reflexive thematic analysis was a further strength which enabled a nuanced exploration and interpretation of participants' lived experiences and subjective realities with interpretive depth. This study also has some limitations that necessitate consideration. Foremost, the sample was recruited online via platforms dedicated to IHMS. As a consequence, those who engage with IHMS outside of the channels were likely excluded. The participants in this study articulated a profound and collective commitment to breastfeeding and had a shared belief in the value of HM. While their narratives provide deep insights in IHMS, their perceptions of CMF cultures may not be representative of the experiences ascribed to the maternity settings in the broader population of maternity care users. Additionally, the sample comprised a homogenous sample of predominantly White Irish, highly‐educated females. This homogeneity may have fostered the strong sense of trust and solidarity observed as a result of having shared cultural, ethnic and social identities which can enhance social cohesion and in‐group trust (Holtug 2025). Additionally, the high levels of trust may have shaped the findings relating to the positive psychological outcomes observed as high trust has been associated with enhanced psychological outcomes (Schneider et al. 2011). Furthermore, the specific geographical context of Ireland may limit the transferability of the findings to international contexts. However, this research from within the island of Ireland makes an important contribution to the wider evidence base on this emerging practice of infant feeding.

## 5 Conclusion

This study represents the first qualitative exploration of IHMS donors and recipients in Ireland. Contextually, this research advances the scholarly understanding of the experiences and practices of IHMS within this national context and while broadening the global discourses of IHMS. Our findings purposefully illuminate the imperatives for IHMS in Ireland. IHMS is fundamentally enabled by an HMB system which is perceived as invisible and replete with obstacles to its engagement. Paradoxically, the perceptions of IHMS participants illuminate the intentional concealment of IHMS and perceived CMF culture within maternity settings. Analogous to this disconnect is the architecture of a “*wee subculture”* where risk and safety are framed as actively assured through a trust‐based system of connection and collective solidarity. Importantly, this study has revealed the significant psychological and emotional impacts for recipients where IHMS was associated with an immediate reduction in recipient distress and anxiety and a sense of pride and fulfillment for donors. Policy efforts should prioritize the pervasive barriers to the universality and accessibility of HMB for both donors and recipients. Simultaneously, the establishment of clinical frameworks for milk sharing must focus on mitigating risk and ensuring the practice is as safe as possible. Future research directions should explore HCP awareness, understanding and knowledge of HMBs and IHMS within Ireland and other underexplored geographical contexts.

## Author Contributions

N.V., A.M. and G.P. all contributed to the conceptualization of the study. N.V. was responsible for data collection, analysis, and drafting the first version of the manuscript. A.M. and G.P. provided critical supervision and oversight, assisting with the design of the study, reviewing data analysis, performing editing and revision of the manuscript. All authors have reviewed and approved the final manuscript.

## Conflicts of Interest

The authors declare no conflicts of interest.

## Data Availability

The data that support the findings of this study are available on request from the corresponding author. The data are not publicly available due to privacy or ethical restrictions.
